# COVID-19 and lockdown schooling: how digital learning environments influence semantic structures and sustainability knowledge

**DOI:** 10.1007/s43621-021-00041-y

**Published:** 2021-07-25

**Authors:** Sonja T. Fiedler, Thomas Heyne, Franz X. Bogner

**Affiliations:** 1 Didactics of Biology, University of Würzburg, Matthias-Lexer-Weg 25 , 97074 Würzburg, Germany; 2 PSE (Professional School of Education), University of Würzburg, Würzburg, Germany; 3 Z-MNU (Centre for Math & Science Education), University of Bayreuth, Bayreuth, Germany

**Keywords:** Education for sustainable development, Digital learning, Lockdown schooling, Sustainability, Semantic differential

## Abstract

Promoting sustainable lifestyles through Education for Sustainable Development (ESD) is part of the UN’s *Agenda 2030*. Earlier empirical studies proved direct interactions with and in natural environments to be effective ESD methods. Pandemic-related lockdowns rendered such courses nearly impossible, which raised concerns about achieving the Sustainable Development Goals (SDGs) in general. To evaluate what young learners know about the concept *sustainability* so far and how it can be taught effectively online, we designed an online learning module tackling sustainability issues and compared it with data from an on-site intervention module for Bavarian 5th graders (~ 10 years old). Cognitive learning as well as attitudinal preferences of 288 learners were monitored in a pretest–posttest design. The learning module comprised two sections: One about botany, plant characteristics, and plant families; the other about the advantages and disadvantages of traditional as well as sustainable farming methods. The customized cognitive test and semantic differentials for *sustainability* and *environmental protection* produced three major findings: (1) A digital learning environment successfully and significantly increased sustainability knowledge (2) Learners clearly distinguished the concepts *Sustainability* and *Environmental Protection* (3) There is no direct correlation between semantic differential scores and learning outcome.

## Introduction

The United Nations’ *Agenda 2030* includes 17 Sustainable Development Goals (SDGs) to address global issues such as climate change, poverty, and inequality. *Education for Sustainable Development* (ESD) is a powerful tool for achieving the SDGs [1]. After the United Nations *Decade of Education for Sustainable Development* started in 2005, ESD has been implemented in syllabi worldwide. Nevertheless, the *Sustainable Development Goals Report 2017*, points out that “… the rate of progress in many areas is far slower than needed to meet the targets by 2030” [2, p. 4]. Singer-Brodowski et al. [3] surveyed the German school system at a national level for ESD elements from 2011 to 2016. They came to the same conclusion that efforts to achieve the SDGs should be increased.

Most ESD initiatives are collaborations between outreach environmental centers and schools. The latter rarely take on the ESD activities by themselves. Hence, there have been huge differences between individual schools in ESD teaching [3]. Obviously, during lockdown schooling visits to such centers have been impossible. A severe setback in ESD may therefore be inevitable and thus failing *Agenda 2030* is foreseeable, too.

### ESD and sustainability

The United Nations defined *sustainability* as “meeting the needs of the present without compromising the ability of future generations to meet their own needs” [4, p. 4]. This rather anthropocentric definition was later modified to include economic, ecological, and social implications [5]. Likewise, *sustainability knowledge* is an interdisciplinary, cross-curricular concept [6]. The UK Department for Environment, Transport and Regions (DETR) defines four key elements of sustainable development: (1) social progress which recognizes the needs of everyone, (2) effective protection of the environment, (3) prudent use of natural resources and (4) maintenance of high and stable levels of economic growth and employment [7]. The UN defines ESD as “empower[ing] learners to transform themselves and the society they live in by developing knowledge, skills, attitudes, competencies and values” [8, p. 7]. They also highlight the interconnectedness of topics such as global citizenship, climate change, and loss of biodiversity. Fully comprehending such topics and their implications is challenging, especially for young learners. Thus, ESD teaching on primary and secondary levels should focus on establishing a solid base of sustainability knowledge before addressing interconnections. The term *sustainable* itself is mostly depicted as synonymous with *environmentally friendly* [9]. Without proper ESD in school, 5th graders are likely to have little to no information about economic and social aspects of sustainability. Thus, young learners are not expected to differentiate between these two terms.

#### H1

5 th graders cannot differentiate between the abstract term sustainability and the more tangible concept of environmental protection.

Several studies have demonstrated that environmentally friendly lifestyles depend on three key factors: knowledge, values, and behavior [10–12]. More precisely, high environmental knowledge scores positively influence pro-environmental attitudes and behaviors [13, 14]. Consequently, establishing in-depth sustainability knowledge will motivate learners to live a more sustainable lifestyle. Keselman et al. [15] describe the discrepancy between the known impact of environmentally friendly actions and the potentially more negative effect of environmentally unfriendly behavior. For example, people may have basic knowledge about biodiversity loss, yet do not care about it unless a viable threat to their lifestyle is revealed, such as shortages in their food supply. Kowasch and Lippe [16] argue that most learners know exactly what sustainable behavior means, but manifest binary perceptions of sustainable lifestyles. Thus, ESD knowledge should first concentrate on everyday life issues in addressees’ context and then move on to a broader scale to avoid such discrepancies. Since young learners have little impact on adult behavior such as buying an e-car or shopping for organic groceries, research on this age group should focus on connections between knowledge and attitudes. Generally, a positive correlation between attitudes and knowledge gain is expected.

#### H2

Positive attitudinal preferences towards sustainability concepts lead to greater learning progress in an ESD module.

To put more emphasis on ESD, the Bavarian Ministry for Education introduced new issues in the curriculum. *Ecosystem Pasture* introduces concepts of sustainability and sustainable practices in the fifth grade. It tackles both economic and ecological aspects. Learning objectives involve plant growth conditions and the impact of agricultural methods on species diversity by contrasting “traditional” farming methods (which focus on the highest yield possible by using fertilizers, insecticides, mechanization of labor) and “sustainable” agriculture (which considers the natural fertility of the land, local climate, etc.). Sustainable agriculture is considered environmentally friendly. These learning objectives go hand in hand with both the UN’s *Global EverGreening Alliance*, the EU’s *Green Deal* and the *From Farm To Fork* initiative [17, 18]. As regenerative farming is regarded as a good way to start environmental education [19] and fits the target groups’ syllabus, it is suitable for this study.

### Digital learning and ESD

Most research on digital ESD has been conducted in higher education [20–22]; however, vast amounts of data are available for online STEM teaching. ESD methodology and STEM teaching share certain similarities: according to Ahel and Lingenau [23] for example both promote problem-solving and cooperative learning strategies to enable learners to tackle future problems. Studies on digital STEM teaching cover a variety of approaches: comparing synchronous and asynchronous approaches, incorporating VR (Virtual Reality) and AR (Augmented Reality) elements, and mobile learning with specific apps and games to name just a few [24–27]. Thompson et al. [28] point out that problem-solving skills are more difficult to train when taught online. Especially during lockdown schooling, online units rarely used collaborative teaching methods, which are essential to ESD [29, 30]. Therefore, less learning progress is expected in an online setting in comparison with a traditional, on-site ESD module.

#### H3

ESD teaching leads to better learning progress when conducted on-site rather than online.

Hodges et al. [31] describe *emergency remote teaching* as different from well-structured, systematic online learning. For example, communication with and feedback by teachers has been sporadic and difficult to obtain, although this is an essential part of effective online learning [32]. Recent studies on lockdown schooling confirmed that teachers as well as learners need training in handling new technologies and digital tools [33, 34]. In normal situations, studies have already investigated the influence of digital learning environments on biology knowledge or environmental attitudes [35]. However, these studies were conducted in schools and monitored by a teacher or supervisor. Apart from asynchronous communication via email, digital teaching as part of blended learning or flipped classroom settings usually relies on personal interaction as well [36]. This means that learners always had some face-to-face time with their teachers besides working on assignments on their own at home. However, lockdown schooling matches elements typical for homeschooling: learners receive standardized input and have specific channels to hand in assignments, but the learning process happens at home without the supervision of a professional educator [37]. As this study was conducted during the first lockdown period, it was designed as asynchronous, with little to no involvement of the teachers.

### Semantic differential as a technique

Semantic Differentiation (Semantic Differential or Differential Semantics) was originally proposed by Osgood, Suci and Tannenbaum [38] in the 1950s as a technique for extracting attitudes toward objects or concepts. The method is now universally applicable to measure any term, object, idea, activity, etc. [39–42]. If, for example, methods such as Likert scales were used in questionnaires, the questions had to fit the respective topic. It is nearly impossible to compare different sets of questions across different topics. It is, however, possible to analyze the semantic structures of different concepts. Thus, Osgood, Suci and Tannenbaum [38] established a universal rating scale based on a standardized set of adjectives. A semantic differential (SD) consists of bipolar adjective pairs, which ideally have an antonymic meaning such as *good* – *bad*. Each set of adjectives is rated on a Likert-type scale. Rosenberg and Navarro [42] recommend eight to 12 bipolar pairs with seven to nine scaling points. The SD method allows an analysis of both the affective and cognitive aspects of the selected concept.

SDs have been used to assess attitudes toward certain topics or objects in a variety of research disciplines, such as social studies, economics, marketing, geology, and architecture [43–49]. It is regarded as an effective psychological tool for measuring multidimensional traits such as personality, attitude, or communication [50]. It also appears to be a promising alternative tool for regular monitoring in schools [51].

Semantic structures and meanings are represented at a multidimensional level as a correlation matrix. This matrix represents the semantic space occupied by the rating of a concept. Each concept is represented by a polarity profile (see Fig. 3), which can be compared to profiles of other respondents toward the same concept or the profile of the same respondent towards other concepts. An initial analysis focuses on the differences between the ratings of binary adjectives. This analysis can also be conducted by comparing the sum scores for each concept. In this study, an SD with a seven-point scale and nine binary pairs was used. Thus, the mean score was 31.5. If a respondent’s sum score was below that number, their attitude toward the concept can generally be summarized as negative, and vice versa.

Further analysis via factor scoring determines the distance between the ratings. Osgood, Suci and Tannenbaum [38] claimed that there are three universal dimensions labeled *evaluation*, *potency*, and *activity*. Through these dimensions, each object or concept can be defined and compared using a specific semantic axis. The three dimensions are widely recognized, yet their factor analysis is rather difficult because they consist of several subscales [40, 41, 45]. Furthermore, critics of a universally applicable SD point out that semantics work differently in cultures and languages [41, 52, 53].

### Research questions

Online learning and ESD are essential components of modern teaching scenarios. Thus, this study aims to evaluate the current status of young learners in both fields contemporaneously, thereby gaining valuable insights for educators and teachers.

1. Which learning progress does a online learning environment for *botany* and *sustainable agriculture* evoke in comparison with an on-site program?
2. To what extent can 5^th^ graders differentiate between *Sustainability* and its subcategory *Environmental Protection* prior to a sustainability module?
3. What correlation can be found between knowledge gain in on-site and online teaching modules and attitudinal preferences measured with a semantic differential?

## Methods and procedures

### Participants

A total of 288 Bavarian 5th graders (10.8 ± 0.45 years, 41% ♀) were recruited with the help of their teachers and the support of the Bavarian Ministry of Education. Participation was voluntary. They completed online questionnaires before and after and intervention-style unit consisting of two separate modules.

### Intervention design and sample

The intervention took place in 2019 and 2020. Two different methods were used to teach curriculum-related content in botany and sustainable agriculture. The control group (CG, n = 86) completed a one-day on-site intervention, whereas the experimental group (EG, n = 202) completed an online asynchronous learning module over the course of two weeks. Both groups received a guidebook referred to as *researcher diary* with tasks either based on information on-site or with links to learning platforms (e.g., Prezi) with customized information.

The first part of the learning unit focused on botanical terms and the categorization of plants. Learners learned to distinguish different plant families for instance by flower shape*.* Besides characterizing several given plants, they also had to go outside and find an unknown plant on their own. The module continued with prerequisites for ideal plant growth. Learners received input about the location factors *humidity*, *temperature*, and *sunlight*. On-site learners were equipped with different measuring tools. Online learners were instructed to build a DIY (do-it-yourself) rain gauge to monitor the humidity of their backyard. The second part continued with growth factors in the context of species diversity. Learners had to gather information about the three location factors in their area, compare them to a given list of plants and select possible new plants to hypothetically plant in their location. Learners were then confronted with the question of why species diversity is a desirable thing in the first place. Among other sources, three expert videos (traditional farmer, sustainable farmer, local politician) with information about traditional and sustainable agriculture were provided. A concluding DIY task was to create a poster of the pros and cons for more sustainable farming in their area. If possible, these posters were presented in class.

### Test design and instruments

Two Semantic Differentials (SD) with nine bipolar pairs and seven scale points were used to assess learners’ attitudes and emotions towards sustainability (see Table 2). The antonyms were partially literature-based and partially expert-selected to suit the target group [42]. See Table 2 for all nine adjective pairs. There was no retest for the SD because previous research has shown that affective dimensions do not change over such a short period [54, 55].

Knowledge levels were monitored through a customized multiple-choice test comprised of 17 items. The items cover botanical knowledge like typical shapes of flowers or the purpose of certain plant organs as well as knowledge about sustainability in agriculture and farming methods (see Table 1). The knowledge test was completed by the learners before they received the learning modules (pretest) and after they completed the last task from the guidebook (posttest). It was not possible to conduct a retention test to assess long-term learning.

### Statistical analysis

IBM SPSS 25.0 was used for factor analysis and Quest for Rasch analysis. Level of significance is marked as p ≤ 0.05 = *; p ≤ 0.01 = **; p ≤ 0.001 = ***. For factor analysis values below 0.3 were left out.

## Results

### Knowledge levels

**Fig. 1:**
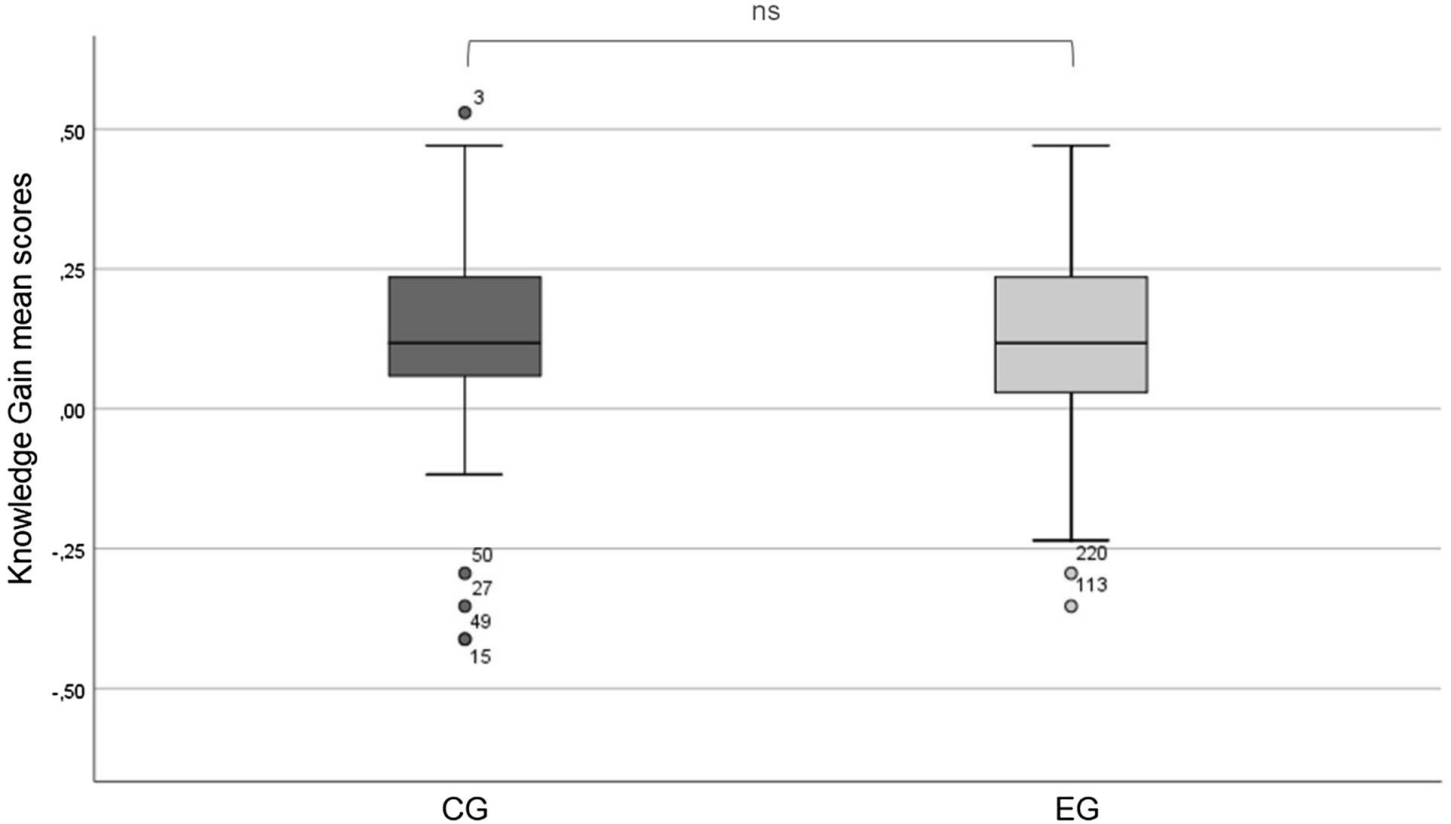
Comparison of knowledge scores in CG and EG

The first part of this analysis focuses on changes in learners’ cognitive perception of the concept *Sustainability*. A comparison of pretest and posttest scores via Wilcoxon test (p < 0.01) showed significant learning progress for both the control group (on-site) and the experimental group (online). Further analysis via Mann–Whitney-U (p > 0.05 at 0.565) tested negative for statistical differences in learning progress between CG and EG (see Fig. 1).

The test itself was analyzed using Rasch. Cronbach’s α assesses internal consistency and was at 0.74 (> 0.7 is good, > 0.8 is very good, > 0.9 is not acceptable; [52, 56]). The Rasch-model-based reliability showed very good item estimates with Infit MNSQ at 0.99 (± 0.08) and Infit t at 0.13 (± 1.62). Especially MNSQ-values are not sensible to sample size and therefore work for small sample groups, too [57]. The reliability of estimate at 0.97 is considered very good and person reliability of 0.63 is also acceptable. Reliability of estimate at 0.98. (± 1.0) and person reliability of 0.55 (± 0.84) are in line with previous analysis that showed the data has no normal distribution. MNSQ for all Item Fits lie between 0.08 and 1.2, which means all items fit the model.

**Fig. 2:**
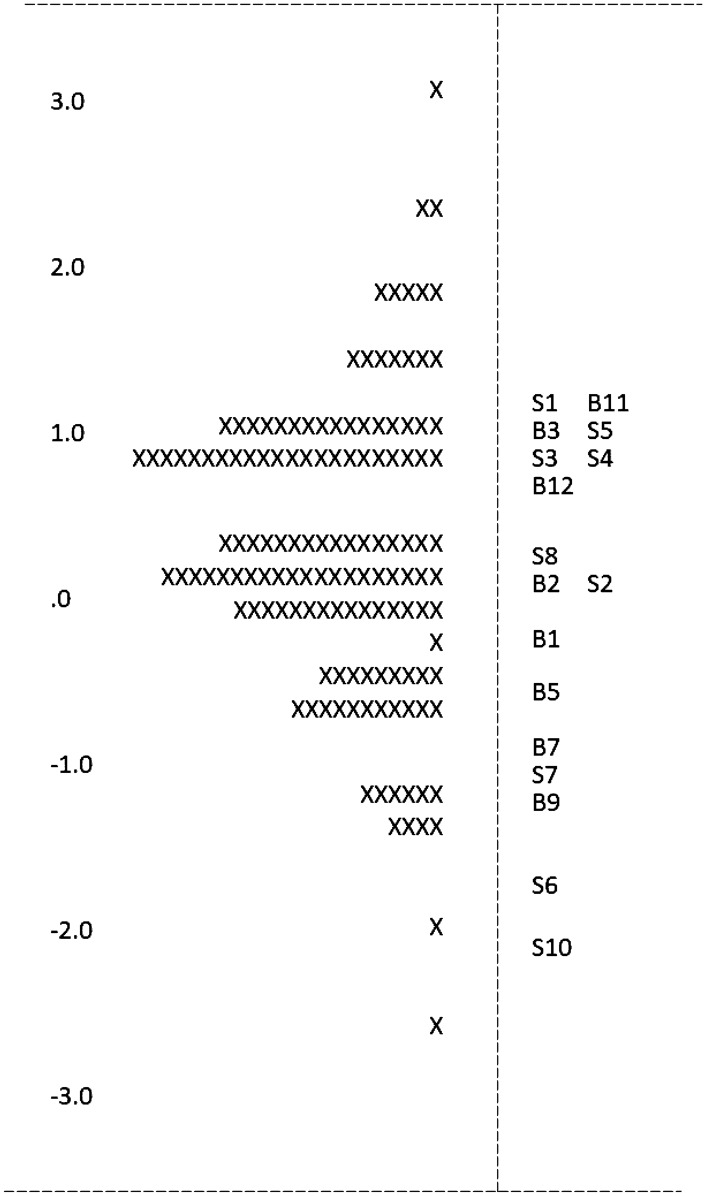
Item estimate threshold for Rasch analysis of knowledge level items. Each X represents 2 learners

Item Estimate Thresholds lie between -3 and + 3 (see Fig. 2). As shown by distribution of the Xs on the left-hand side, cases are spread evenly. The grouping of the questions (Bs and Ss on the right-hand side) indicates that there are no very hard items, many moderate-to-hard items, and one easy item (S10). Therefore, reaching a satisfactory score on the test was fairly easy, but only few learners reached close-to-perfect scores. Several example questions (see Table 1) display a mix of easy, moderate, and hard questions.

**Table 1:** Example questions for the knowledge test with example answer options for two questions

B12	Here are two pictures of cherry tree flowers. To which plant family does the cherry belong to?	
	❏ Mint family	❏ Bean family
	❏ Rose family	❏ Cabbage family
S11	Which of the following farming techniques are used in traditional agriculture?	
S1	The term “sustainability” is closely related to how we use natural resources. Which of the following statements explains a sustainable use of natural resources the best?	
B9	The flower of labiate plants consists of …	
B11	You want to find the ideal place for a beehive. Which environment holds the ideal source of nutrition for bees?	
	❏ Cornfield	❏ Flower meadow
	❏ Soccer pitch	❏ Deciduous forest

### Semantic differential

**Fig. 3:**
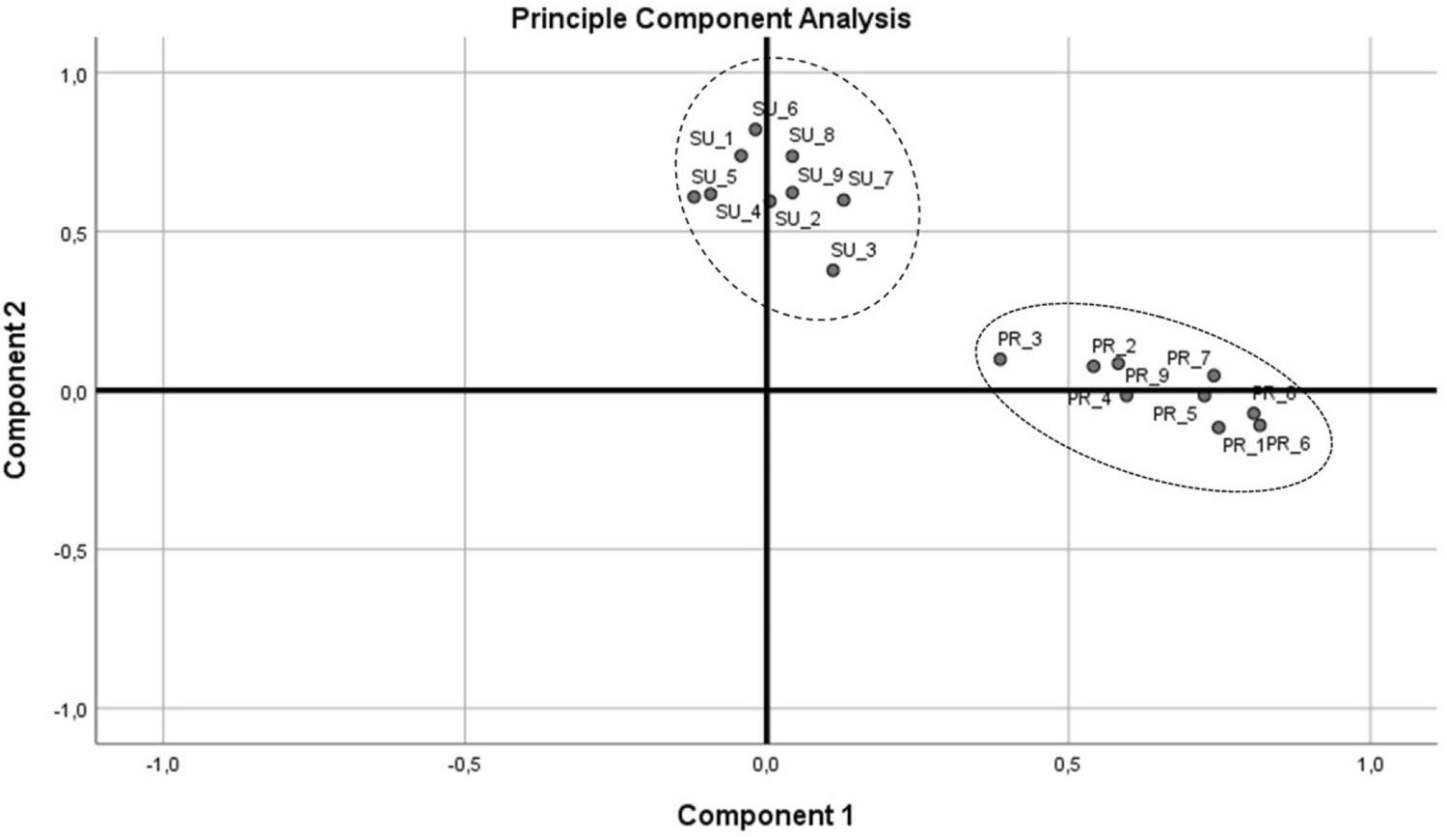
Semantic Differential Scores of PR and SU for all adjective pairs

The second part of this analysis focuses on two semantic differentials, one for *Sustainability* (SU) and the other one for *Environmental Protection* (PR). Wilcoxon test at 0.086 showed no significant difference between scores in PR and SU. Mean score for PR was at 5.72 (± 0.67) slightly lower than for SU at 5.85 (± 0.71). The adjective pair *hard-easy* had the lowest ranking, whereas *useless-useful* showed the highest ranking (see Fig. 3).

**Fig. 4:**
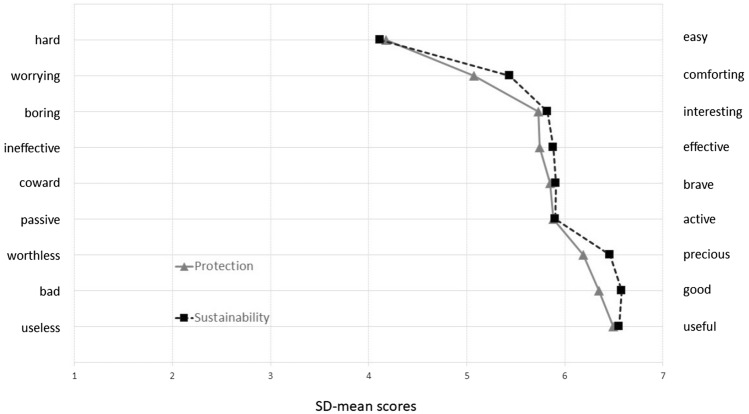
Principal Component Analysis of both Semantic Differentials

The SDs then were analyzed using Principal Component Analysis (PCA) with Oblimin rotation to validate the basic structure proposed by Osgood, Suci and Tannenbaum [38]. KMO for PCA was tested adequate at 0.804. (Kaiser–Meyer–Olkin is considered > 0.7 as intermediate, > 0.8 as good, > 0.9 as very good [58];). Bartlett’s test of sphericity (χ^2^ = 1559.099, p < 0.001) indicated that correlations between items were sufficiently large for PCA. As illustrated by Fig. 4, loading scores of individual adjective pairs showed two principal components: one component consists of all PR-Couples and the other one of all SU-couples.

A second PCA with Oblimin rotation was applied on each individual set of bipolar pairs. KMO verified sampling adequacy (KMO = 0.813, χ^2^ = 671,737, p < 0.001). This analysis was conducted to check the data for the three underlying dimensions *evaluation*, *potency*, and *activity*. Three adjective pairs matched each of the dimensions (see Table 2).

**Table 2:** SD SU factor scores of PCA analysis with Oblimin rotation

Components	Evaluation	Potency	Activity
Bad—good	.908		
Useless—useful	.893		
Worthless—precious	.771		
Hard—easy		.898	
Ineffective—effective		.552	
Worrying—comforting		.522	
Passive—active			.884
Coward—brave			.836
Boring—interesting			.386

After the dimensions had been verified the analysis continued as Heise [59] suggested with calculating the distance between the two concepts for each dimension (e.g. distance for *Evaluation* d_E_ = √(E_1_-E_2_)^2^). The results were d_E_ = 0.187, d_P_ = 0.15, d_A_ = 0.057. Higher scores indicate greater dissimilarity between the concepts. None of the distance scores is above 1.0 though, so they can be considered rather close overall. This falls in line with previous Wilcoxon test.

### Correlation between semantic structure and knowledge levels

**Fig. 5:**
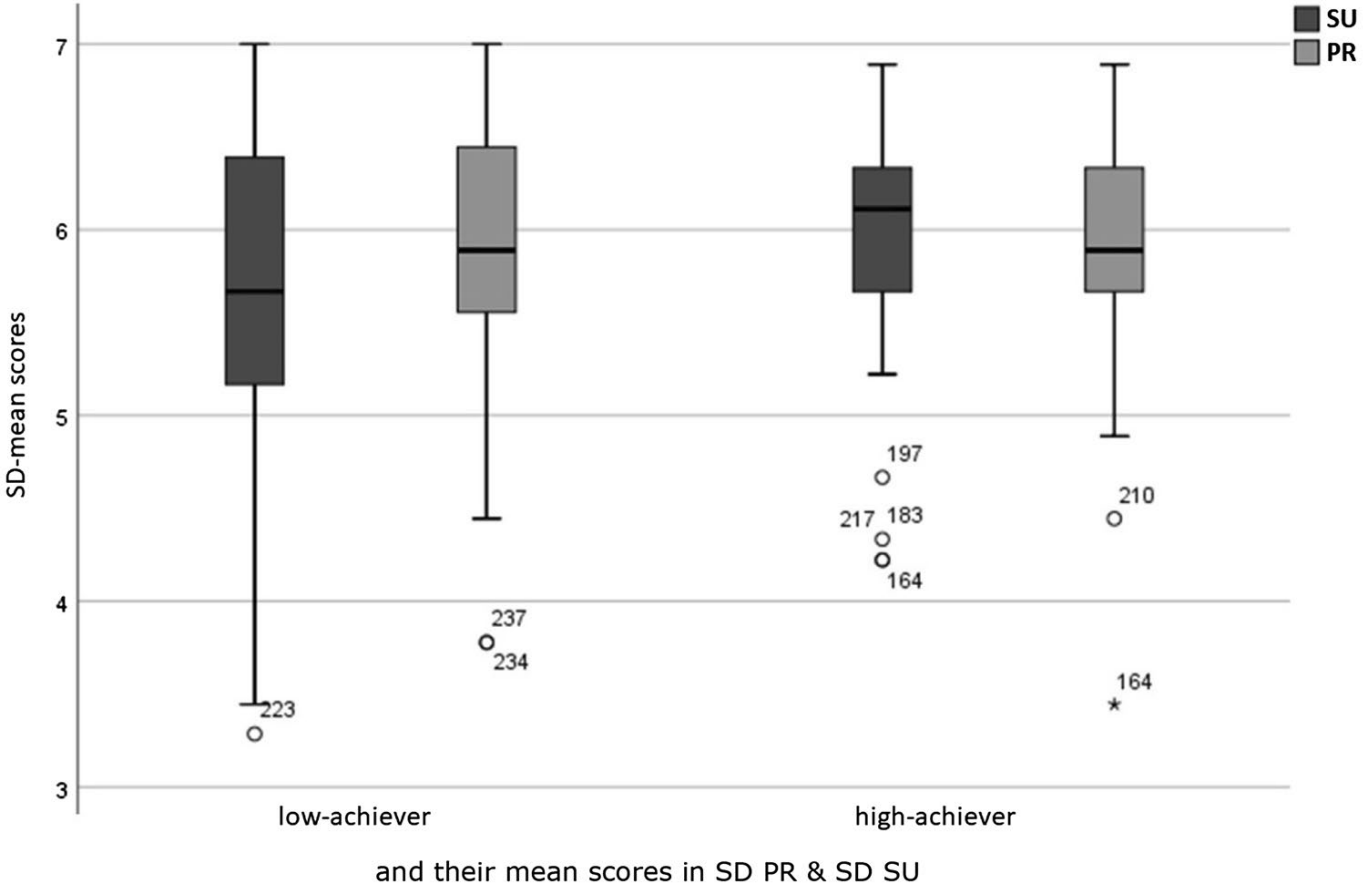
Comparison of knowledge levels (high- and low-achievers) and their mean scores in SD PR & SD SU

Finally, correlations between posttest knowledge scores and SD scores were calculated. High-achievers (with knowledge scores in the posttest above median) and low-achievers were compared. Spearman-Rho analysis resulted in significant positive correlations between scores in SD-PR and SD-SU (0.598**). Mann–Whitney-U test showed no significant difference between low-achiever or high-achiever mean SD scores (0.13 for SD SU and 0.72 for SD PR). Mean Scores for SD-SU were 5.64 ± 0.86 for low-achievers and 5.91 ± 0.71 for high-achievers. Mean Scores for SD-PR were 5.93 ± 0.71 for low-achievers and 5.87 ± 0.7 for high-achievers (see Fig. 5).

## Discussion

A major surprise is the same learning outcomes of online and on-site interventions. Previous studies have found that outdoor activities are more effective for sustainability knowledge acquisition [54, 55, 60, 61]. Due to the unique situation caused by COVID-19 lockdown schooling, learners could not engage in outdoor learning activities. It provided an exceptional opportunity to survey children who had not been exposed to didactically prepared natural environments during the study. Contrary to Hypothesis 3, our findings clearly indicate that even a sole digital learning environment significantly alters knowledge levels. This supports studies like Schönfelder and Bogner [62], where no significant difference between on-site and online learning was found. Also [23], point out that asynchronous online learning has some positive practical implications for learners: online content is easily accessible and fits flexibly into learners’ daily routines. In an on-site intervention, however, the learning environment is more controlled and more insight, for example, into the learners’ motivation is possible. This asynchronous approach was driven by an unprecedented lockdown learning situation. A new approach using blended learning can increase the comparability of on-site and online studies. Other key elements of ESD, such as questioning and re-imagining conventional, non-sustainable lifestyles and empowering learners to adopt more community action [30], could benefit from using more collaborative learning forms.

Since the knowledge test had to be customized to fit the intervention’s contents, applying Rasch analysis was advisable to assess its model fit. The items tended to be easy, but the cases were spread evenly, and fit indices were within suitable ranges. Internal consistency values were also considered to be good [63, 64]. Therefore, the applied knowledge test did indeed account for botanical and environmental knowledge. Both the experimental group (online) and the control group (on-site) had a significant increase in knowledge. Although items were moderate to easy, there were no perfect scores for either of them. Since knowledge gains were comparable, the age group would seem to have significant influence on this outcome. [65] suggest that 5^th^ graders tend to have high learning motivation in science. *Ecosystem pasture* is the first unit in the Bavarian curriculum to target ESD. So, pre-knowledge levels would be rather low compared to other topics. Research indicated that pre-knowledge must be considered if present and that knowledge levels increase more easily from low to moderate scores than moderate to high scores [65, 66].

Our second major finding is that 5th graders can differentiate between the two concepts in question as shown by the PCA of both SD values. Our results point to a distinct differentiation between the abstract concept of *Sustainability (SU)* and the more tangible concept of *Environmental Protection (PR)*. Most studies focus on the latter while others do not differentiate between ecological, economic, and social aspects [29]. Our semantic structure shows that 10-year-olds can distinguish the complex concept of sustainability from protecting natural environments. Hypothesis 1 thus has to be rejected. To avoid contradictions between sustainability knowledge and actual behavior as suggested by Kowasch and Lippe [16], future research on children’s attitudinal preferences towards economic and social aspects of sustainability is recommended. As the UN framework points out, the interconnectedness of ESD-related phenomena is key to promoting more sustainable lifestyles. Learners’ knowledge should therefore not be limited to one subject. Our Grade 5 learning units successfully targeted both economic and ecological effects of sustainable and traditional farming. Thus, interdisciplinary topics are suitable for ESD on the secondary levels.

The three-dimensional structure (evaluation, potency, and activity) of semantic differentials has been questioned by a variety of studies [46, 47, 67]. Maclay and Ware [53] point out that semantics work differently in different cultural contexts. According to Chráska and Chrásková [68] this must be considered when using the SD method. Therefore, bipolar adjective pairs need to be standardized for a specific cultural environment [41]. Our adjective pool consists of pairs suggested by previous research as well as additional pairs considered suitable for this age group by experts [40, 42]. The PCA showed all three categories. Hence, our mix of inductive and deductive techniques when choosing appropriate bipolar pairs may be regarded as successful. Consequently, the SD method proved to be an easily applied, convenient method to tackle a variety of research questions – provided that the adjective pairs are representative.

As other statistical tests already showed no significant difference between the affective space of *Sustainability* and *Protection*, it was likely that the three subcategories did not differ significantly. A comparison of these findings with the first PCA (see Fig. 3), however, revealed some interesting implications. On the one hand, learners can clearly distinguish between both concepts. On the other hand, they are rated almost the same, although *Sustainability* is rated slightly higher. *Environmental protection* is a part of the ecological and economic pillars of *sustainability*. Acting sustainability-conscious, therefore, automatically includes actions toward environmental protection. The least-rated item in both SDs was *hard-easy*. This shows a rather ambivalent kind of thinking within the 5^th^ graders: apparently, they know that acting sustainably is important since *good* and *precious* have the highest ranking, but they also evaluate it as a rather difficult task to accomplish. In this context, it would be interesting to track social desirability scores with comparable sample groups [69–71]. Young learners can lack the foresight needed to evaluate the consequences of more sustainable behavior. Prospectively then, SD values could drop once learners were informed about the downsides of sustainable development, such as increased food prices.

Finally, the influence of attitudinal preferences on learning outcomes was analyzed. Since there was no significant difference between the CG and EG, we combined both groups for this analysis. A comparison of high and low achievers, the ones that learned the most and the least during the intervention, showed no statistically significant difference between SD ratings and learning success. This contradicts Hypothesis 2 and previous research [11, 13, 72]. However, Fig. 5 shows a greater standard deviation for SD-SU in low achievers than in high achievers. These findings lead to the possible conclusion that learners, who may have had no idea what the abstract term *sustainability* meant before the intervention, were simply checking boxes. Hence, some ratings were rather poor, while others were higher as expected. One possible explanation for this increased deviation could be that the term *environmental protection* is used more commonly and is more self-explanatory. Therefore, learners were much more confident about how to rate this concept. Since the SD was collected before the intervention, *sustainability* had not yet appeared in the official syllabus. Therefore, they had either no pre-knowledge about it or possibly biased sources, such as their parents or social media.

This is especially interesting for educators in ESD. Sammalisto et al. [22] point out, specific courses on sustainability can influence university learners’ perceived environmentally friendly behavior. Our findings, however, indicate that educators in younger grades should ensure that there is a certain common ground of sustainability knowledge to build upon before commencing with detailed knowledge. Middle school teachers can assume that there is no common knowledge basis in their classes. Thus, before tackling popular issues such as plastic waste or conducting field courses, learners should first establish an adequate theoretical background about sustainability. In a blended learning scenario, this basic knowledge should be taught through asynchronous online activities, whereas synchronous learning units should be used for direct interaction with and in natural environments.

### Conclusion

Surprisingly, our ESD module led to similar learning progress online and on-site. Contrary to studies in environmental education, attitudinal preferences measured before the learning module had no significant impact on knowledge gains. Gathering more information about social desirability in this age group could shed more light on their attitudinal preferences toward sustainability concepts in general. The semantic differential method was used to measure these preferences. This is especially valuable for teachers and educators, as using adjective pairs is a rather easy design for a test instrument and offers a short but effective monitoring tool. Based on the convenience of the SD tool, it should find its way to regular classroom practice. However, testing the SD method for other concepts and different age groups could provide further insight into its universal application.

Since sustainable agriculture touches many everyday issues even for learners, such as choice of food or production method, it is a suitable starting point for ESD. The topic is not limited to ecologic aspects but provides a variety of possible expansions toward other SDGs, such as political agendas or economic implications. As shown by our analysis of online distance learning, lockdown schooling did not necessarily cause a setback to the SDGs. However, further research with a more representative sample size is required. Other digital teaching methods such as blended learning and collaborative modules should also be evaluated for primary and secondary level teaching.

## Data Availability

Data cannot be shared publicly because of the Bavarian Ministry for Education's guidelines regarding data of underaged participants. Data are available for researchers who meet the criteria for access to confidential data. Access must be confirmed by the Ethics Committee of the University of Bayreuth/ ZMNU. Contact the corresponding author for further information.

## References

[1] Rieckmann M. Education for Sustainable Development Goals: Learning Objectives. UNESCO Publishing; 2017.

[2] UNSD. The sustainable development goals report 2017. New York: UN; 2017.

[3] Singer-Brodowski M, Brock A, Etzkorn N, et al. Monitoring of education for sustainable development in Germany – insights from early childhood education, school and higher education. Environ Educ Res. 2019;25:492–507. 10.1080/13504622.2018.1440380.

[4] Brundtland GH. Report of the World Commission on Environment and Development: Our Common Future. WCED; 1987.

[5] Imran S, Alam K, Beaumont N. Reinterpreting the definition of sustainable development for a more ecocentric reorientation. Sust Dev. 2014;22:134–44. 10.1002/sd.537.

[6] Sund P, Gericke N. Teaching contributions from secondary school subject areas to education for sustainable development—a comparative study of science, social science and language teachers. Environ Educ Res. 2020;26:772–94. 10.1080/13504622.2020.1754341.

[7] DETR Report. Sustainable development: What it is and what you can do? DETR Green Ministers Report; 2000.

[8] UNESCO. Framework for the implementation of Education for Sustainable Development (ESD) beyond 2019. Paris: UNESCO General Conference; 2019.

[9] Salovaara JJ, Pietikäinen J, Cantell H. Perceptions of interconnected sustainability: Students’ narratives bridging transition and education. J Clean Prod. 2021;281: 125336. 10.1016/j.jclepro.2020.125336.

[10] Geiger SM, Dombois C, Funke J. The role of environmental knowledge and attitude: Predictors for ecological behavior across cultures. An analysis of argentinean and german students. Umweltpsychologie. 2018;22: 69–87.

[11] Faize FA, Akhtar M. Addressing environmental knowledge and environmental attitude in undergraduate students through scientific argumentation. J Clean Prod. 2020;252: 119928. 10.1016/j.jclepro.2019.119928.

[12] Roczen N, Kaiser FG, Bogner FX, et al. A Competence model for environmental education. Environ Behav. 2014;46:972–92. 10.1177/0013916513492416.

[13] Liefländer AK, Bogner FX. Educational impact on the relationship of environmental knowledge and attitudes. Environ Educ Res. 2018;24:611–24. 10.1080/13504622.2016.1188265.

[14] Maurer M, Koulouris P, Bogner FX. Green awareness in action—how energy conservation action forces on environmental knowledge, values and behaviour in adolescents’ school life. Sustainability. 2020;12:955. 10.3390/su12030955.

[15] Keselman A, Levin DM, Hundal S, et al. Teaching environmental health science for informed citizenship in the science classroom and afterschool clubs. Int J Sci Soc. 2012;3:31–44. 10.18848/1836-6236/CGP/v03i03/51346. 24382985 PMC3875328

[16] Kowasch M, Lippe DF. Moral impasses in sustainability education? Empirical results from school geography in Austria and Germany. Environ Educ Res. 2019;25:1066–82. 10.1080/13504622.2018.1557112.

[17] European Commission. From Farm To Fork: The European Green Deal, 2019. https://eur-lex.europa.eu/resource.html?uri=cellar:b828d165-1c22-11ea-8c1f-01aa75ed71a1.0002.02/DOC_1&format=PDF

[18] United Nations. Report on the 2019 UN Climate Action Summit; 2019. https://www.un.org/sites/un2.un.org/files/cas_report_11_dec_0.pdf.

[19] Burns EA. Placing regenerative farming on environmental educators’ horizons. Austr J Environ Educ. 2020. 10.1017/aee.2020.21.

[20] Caird S, Lane A, Swithenby E, et al. Design of higher education teaching models and carbon impacts. Int J of Sus in Higher Ed. 2015;16:96–111. 10.1108/IJSHE-06-2013-0065.

[21] Li C, Zhou H. Enhancing the efficiency of massive online learning by integrating intelligent analysis into MOOCs with an application to education of sustainability. Sustainability. 2018;10:468. 10.3390/su10020468.

[22] Sammalisto K, Sundström A, von Haartman R, et al. Learning about sustainability—what influences students’ self-perceived sustainability actions after undergraduate education? Sustainability. 2016;8:510. 10.3390/su8060510.

[23] Ahel O, Lingenau K. Opportunities and Challenges of Digitalization to Improve Access to Education for Sustainable Development in Higher Education. In: Filho WL (ed) Universities as living labs for sustainable development: Supporting theimplementation of the sustainable development goals. Springer; 2020: 341–356. 10.1007/978-3-030-15604-6

[24] Wang A, Thompson M, Roy D, et al. Iterative user and expert feedback in the design of an educational virtual reality biology game. Interactive Learn Environ. 2019. 10.1080/10494820.2019.1678489.

[25] Dehghani M, Mohammadhasani N, Hoseinzade Ghalevandi M, et al. Applying AR-based infographics to enhance learning of the heart and cardiac cycle in biology class. Interactive Learn Environ. 2020. 10.1080/10494820.2020.1765394.

[26] Laru J, Järvelä S, Clariana RB. Supporting collaborative inquiry during a biology field trip with mobile peer-to-peer tools for learning: a case study with K-12 learners. Interact Learn Environ. 2012;20:103–17. 10.1080/10494821003771350.

[27] Fiedler ST, Heyne T, Bogner FX. Explore your local biodiversity—how school grounds evoke visions of sustainability. Am Biol Teach. 2020;82:606–13. 10.1525/abt.2020.82.9.606.

[28] Thompson KV, Nelson KC, Marbach-Ad G, et al. Online interactive teaching modules enhance quantitative proficiency of introductory biology students. CBE Life Sci Educ. 2010;9:277–83. 10.1187/cbe.10-03-0028. 20810959 10.1187/cbe.10-03-0028 PMC2931674

[29] Watson MK, Pelkey J, Noyes C, et al. Using Kolb’s learning cycle to improve student sustainability knowledge. Sustainability. 2019;11:4602. 10.3390/su11174602.

[30] Sinakou D, Pauw B, et al. Designing powerful learning environments in education for sustainable development: a conceptual framework. Sustainability. 2019;11:5994. 10.3390/su11215994.

[31] Hodges C, Moore S, Lockee B, Trust T, Bond A. The difference between emergency remote teaching and online learning. Educause Rev. 2020;1–12.

[32] Nieto-Márquez NL, Baldominos A, Pérez-Nieto MA. Digital teaching materials and their relationship with the metacognitive skills of students in primary education. Education Sciences. 2020;10:113. 10.3390/educsci10040113.

[33] Sáiz-Manzanares MC, Marticorena-Sánchez R, Muñoz-Rujas N, et al. Teaching and learning styles on moodle: an analysis of the effectiveness of using STEM and Non-STEM Qualifications from a Gender Perspective. Sustainability. 2021;13:1166. 10.3390/su13031166.

[34] Triviño-Cabrera L, Chaves-Guerrero EI, Alejo-Lozano L. The figure of the teacher-prosumer for the development of an innovative, sustainable, and committed education in times of COVID-19. Sustainability. 2021;13:1128. 10.3390/su13031128.

[35] Yang K-T, Wang T-H, Chiu C. Study the effectiveness of technology-enhanced interactive teaching environment on student learning of junior high school biology. J Math Sci Technol Educ. 2015. 10.12973/eurasia.2015.1327a.

[36] Chen F, Lui AM, Martinelli SM. A systematic review of the effectiveness of flipped classrooms in medical education. Med Educ. 2017;51:585–97. 10.1111/medu.13272. 28488303 10.1111/medu.13272

[37] van der Keylen P, Lippert N, Kunisch R, et al. Asynchronous, digital teaching in times of COVID-19: a teaching example from general practice. GMS J Med Educ. 2020;37:98. 10.3205/zma001391. 10.3205/zma001391 PMC7740025 33364377

[38] Osgood CE, Suci GJ, Tannenbaum PH. The measurement of meaning. Urbana-Champaign: University of Illinois Press; 1978.

[39] Friborg O, Martinussen M, Rosenvinge JH. Likert-based vs. semantic differential-based scorings of positive psychological constructs: a psychometric comparison of two versions of a scale measuring resilience. Personality Individ Differ. 2006;40:873–84. 10.1016/j.paid.2005.08.015.

[40] Ploder A, Eder A. Semantic Differential. In: Wright JD, editor. International encyclopedia of the social & behavioral sciences. 2nd ed. Amsterdam: Elsevier; 2015. p. 563–71.

[41] Stoklasa J, Talášek T, Stoklasová J. Semantic differential for the twenty-first century: scale relevance and uncertainty entering the semantic space. Qual Quant. 2019;53:435–48. 10.1007/s11135-018-0762-1.

[42] Rosenberg B, Navarro MA. Semantic differential scaling. In: Frey BB (ed) Educational reserach, measurement and evaluation. Los Angeles: SAGE; 2018;140-44.

[43] Marinelli N, Fabbrizzi S, Alampi Sottini V, et al. Generation Y, wine and alcohol. A semantic differential approach to consumption analysis in Tuscany. Appetite. 2014;75:117–27. 10.1016/j.appet.2013.12.013. 24370355 10.1016/j.appet.2013.12.013

[44] Dal Palù D, Buiatti E, Puglisi GE, et al. The use of semantic differential scales in listening tests: a comparison between context and laboratory test conditions for the rolling sounds of office chairs. Appl Acoust. 2017;127:270–83. 10.1016/j.apacoust.2017.06.016.

[45] Papendick M, Bohner G. “Passive victim - strong survivor”? Perceived meaning of labels applied to women who were raped. PLoS ONE. 2017;12: e0177550. 10.1371/journal.pone.0177550. 28493976 10.1371/journal.pone.0177550 PMC5426776

[46] Klettner S. Affective communication of map symbols: a semantic differential analysis. Int J Geo-Inform. 2020;9:289. 10.3390/ijgi9050289.

[47] Zhao Z, Ren J, Wen Y. Spatial perception of urban forests by citizens based on semantic differences and cognitive maps. Forests. 2020;11:64. 10.3390/f11010064.

[48] Puyana-Romero V, Maffei L, Brambilla G, et al. Sound water masking to match a waterfront soundscape with the users’ expectations: the case study of the Seafront in Naples Italy. Sustainability. 2021;13:371. 10.3390/su13010371.

[49] Radulescu CV, Ladaru G-R, Burlacu S, et al. Impact of the COVID-19 Pandemic on the Romanian Labor Market. Sustainability. 2021;13:271. 10.3390/su13010271.

[50] Margono G. Multidimensional reliability of instrument for measuring students’ attitudes toward statistics by using semantic differential scale. Am J Educ Res. 2015;3:49–53.

[51] Stöckert A, Bogner FX. Cognitive learning about waste management: how relevance and interest influence long-term knowledge. Education Sciences. 2020;10:102. 10.3390/educsci10040102.

[52] Streiner DL. Starting at the beginning: an introduction to coefficient alpha and internal consistency. J Pers Assess. 2003;80:99–103. 10.1207/S15327752JPA8001_18. 12584072 10.1207/S15327752JPA8001_18

[53] Maclay H, Ware EE. Cross-cultural use of the semantic differential. Behav Sci. 1961;6:185–90. 10.1002/bs.3830060303. 13764948 10.1002/bs.3830060303

[54] Braun T, Dierkes P. Connecting students to nature – how intensity of nature experience and student age influence the success of outdoor education programs. Environ Educ Res. 2017;23:937–49. 10.1080/13504622.2016.1214866.

[55] Boeve-de Pauw J, van Hoof J, van Petegem P. Effective field trips in nature: the interplay between novelty and learning. J Biol Educ. 2019;53:21–33. 10.1080/00219266.2017.1418760.

[56] Bland JM, Altman DG. Cronbach’s alpha. BMJ. 1997;314:572. 10.1136/bmj.314.7080.572. 9055718 10.1136/bmj.314.7080.572 PMC2126061

[57] Bond T, Yan Z, Heene M. Applying the Rasch Model. Routledge; 2020.

[58] Kaiser HF. A second generation little jiffy. Psychometrika. 1970;35:401–15. 10.1007/BF02291817.

[59] Heise DR. The semantic differential and attitude research. Attitude Measurement. 1970;4:235–53.

[60] Gómez-Ruiz M-L, Morales-Yago F-J, de Lázaro-Torres M-L. Outdoor education, the enhancement and sustainability of cultural heritage: medieval Madrid. Sustainability. 2021;13:1106. 10.3390/su13031106.

[61] Mullenbach LE, Andrejewski RG, Mowen AJ. Connecting children to nature through residential outdoor environmental education. Environ Educ Res. 2019;25:365–74. 10.1080/13504622.2018.1458215.

[62] Schönfelder ML, Bogner FX. Two ways of acquiring environmental knowledge: by encountering living animals at a beehive and by observing bees via digital tools. Int J Sci Educ. 2017;39:723–41. 10.1080/09500693.2017.1304670.

[63] Oon P-T, Fan X. Rasch analysis for psychometric improvement of science attitude rating scales. Int J Sci Educ. 2017;39:683–700. 10.1080/09500693.2017.1299951.

[64] Huang F, Huang L, Oon P-T. Constructs Evaluation of Student Attitudes Toward Science—A Rasch Analysis. In: Khine MS, editor. Rasch Measurement: Applications in Quantitative Educational Research. Singapore: Springer; 2020. p. 139–57.

[65] Hailikari T, Katajavuori N, Lindblom-Ylanne S. The relevance of prior knowledge in learning and instructional design. Am J Pharm Educ. 2008;72:113. 10.5688/aj7205113. 19214267 10.5688/aj7205113 PMC2630138

[66] Filho PS. Identifying students’ prior knowledge to enable Meaningful Learning. IJAERS. 2021;8:273–7. 10.22161/ijaers.84.32.

[67] Llinares C, Page A. Analysis of gender differences in the perception of properties: an application for differential semantics. J Ind Eng Manag. 2009. 10.3926/jiem.2009.v2n1.p273-298.

[68] Chráska M, Chrásková M. Semantic differential and its risks in the measurement of students’ attitudes. Procedia Soc Behav Sci. 2016;217:820–9. 10.1016/j.sbspro.2016.02.155.

[69] Camerini A-L, Schulz PJ. Social desirability bias in child-report social well-being: evaluation of the children’s social desirability short scale using item response theory and examination of its impact on self-report family and peer relationships. Child Indic Res. 2018;11:1159–74. 10.1007/s12187-017-9472-9.

[70] Roth M, Altmann T. A multi-informant study of the influence of targets’ and perceivers’ social desirability on self-other agreement in ratings of the HEXACO personality dimensions. J Res Pers. 2019;78:138–47. 10.1016/j.jrp.2018.11.008.

[71] Verardi S, Dahourou D, Ah-Kion J, et al. Psychometric properties of the marlowe-crowne social desirability scale in eight African Countries and Switzerland. J Cross Cult Psychol. 2010;41:19–34. 10.1177/0022022109348918.

[72] Bradley JC, Waliczek TM, Zajicek JM. Relationship between environmental knowledge and environmental attitude of high school students. J Environ Educ. 1999;30:17–21. 10.1080/00958969909601873.

